# Digital Pathology in Dermatology – Current Status, Applications and Perspectives

**DOI:** 10.1111/ddg.70482x

**Published:** 2026-08-04

**Authors:** Cornelia S. L. Müller, Torsten Hansen

**Affiliations:** ^1^ Medical Service Center for Histology Cytology and Molecular Pathology Trier, Germany; ^2^ Saarland University Medical Faculty Homburg/Saar Germany

**Keywords:** Artificial intelligence, Digital pathology, Dermatopathology, Quality assurance, Telepathology, Whole‐slide imaging

## Abstract

Digital pathology has become an increasingly established component of routine diagnostic practice in recent years. Whole‐slide imaging enables the complete digitization of histological slides and allows for primary diagnosis using digital images. This development offers new opportunities for diagnostics, consultations, archiving, education, and quality assurance. In dermatology and dermatopathology, digital pathology is particularly relevant for the evaluation of inflammatory skin diseases, melanocytic lesions, epithelial skin tumors, and immunohistochemical analyses. This article provides an overview of the technical principles of digital pathology, describes workflow integration and implementation in clinical routine, and outlines requirements for quality assurance and validation. Key applications in dermatology as well as advantages and limitations of digital diagnosis are discussed. A dedicated section addresses the role of artificial intelligence as an assistive tool in dermatopathological diagnostics. The aim of this CME article is to provide dermatologists and pathologists with a comprehensive basis for evaluating and applying digital pathology systems in clinical practice and to place current developments into a realistic clinical context.

## INTRODUCTION

In recent years, digital pathology has developed from a primarily technical innovation to an established component of routine diagnostic practice. Especially the introduction of whole‐slide imaging (WSI), enabling digitization and evaluation of complete histological slides in high resolution on a computer monitor, provides new opportunities for diagnostics, consultation, education, and quality assurance.^1^, ^2^ At the same time, the use of computational image analysis and artificial intelligence (AI) has become considerably more important in pathology.^3^

Digital pathology has particular potential in dermatology and dermatopathology. The high morphological diversity of inflammatory dermatoses, melanocytic lesions, and epithelial skin tumors places high demands on experience, comparability, and interdisciplinary exchange. Digital slides enable location‐independent diagnosis, facilitate dermatopathological consultations, and support standardized archiving of histological findings. Several studies on the use of WSI in dermatopathology show a high level of diagnostic agreement between digital diagnosis and conventional diagnosis by microscopic examination of glass slides if adequate technical quality and structured validation are ensured.^4^, ^5^

Despite the increasing availability of digital systems, challenges still exist with respect to implementation, workflow integration, and legal frameworks. The transition from microscopic diagnosis to primarily digital diagnosis requires careful planning taking scanner technique, image quality, IT infrastructure, data storage, and data protection into account. International experience demonstrates that successful implementation of digital pathology systems depends significantly on standardized validation processes and gradual integration into routine laboratory operations.^6^, ^7^

Another focus of the current development is the use of AI‐based assistance systems. These applications are used, in particular, for detection, classification, and quantification of histological structures and may support the medical diagnosis. Systematic reviews and meta‐analyses demonstrate that AI‐based procedures can reach high diagnostic performance in selected applications, although there are limitations with respect to generalizability, data quality, and clinical validation.^8^, ^9^ AI is currently used complementary to medical diagnosis and does not replace it.^3^

The aim of this CME article is to provide a structured overview of the basic aspects of digital pathology, present relevant applications in dermatology, and place its opportunities and limitations into a realistic context. Apart from technical and organizational aspects, quality assurance, validation strategies, and future perspectives are addressed. This article is intended to provide dermatologists and pathologists with a comprehensive basis for the evaluation and proper use of digital pathology systems in clinical routine.

Digital pathology refers to the digitization of complete histological slides for primarily digital diagnosis on a computer monitor.

## TECHNICAL PRINCIPLES OF DIGITAL PATHOLOGY

**FIGURE 1:**
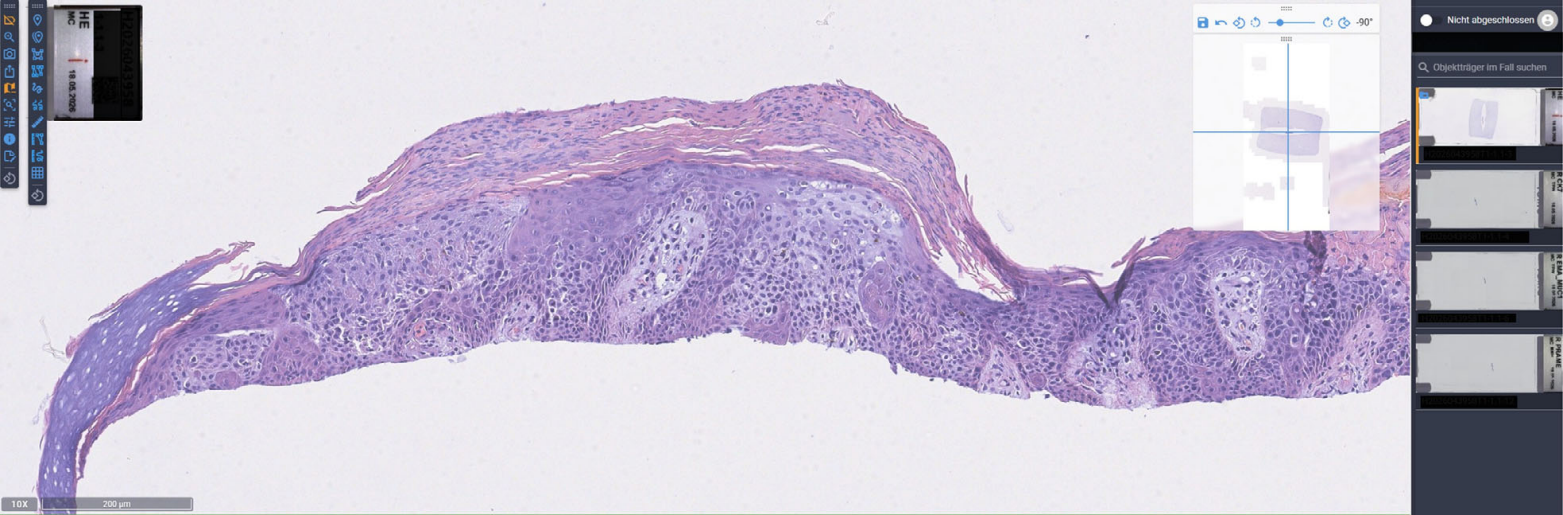
Example of a digitized histological specimen (whole‐slide image) displayed in a digital pathology viewer.

Digital pathology is based on the complete digitization of histological slides by so‐called whole‐slide imaging (WSI). In this approach, conventional glass slides are captured by special scanners in high resolution and stored as digital total images allowing for their microscopic evaluation on a computer monitor^1^, ^2^ (Figure 1). In contrast to static individual exposures, WSI enables continuous navigation through the whole slide at different magnification levels, thus forming the basis for a primarily digital diagnosis.

### Whole‐slide imaging and scanner technology

Typically, WSI scanners acquire histological sections at an optical magnification of 20× or 40× corresponding to an effective resolution in the submicron range. Modern systems have automatic algorithms for focus and exposure to guarantee consistent image quality across the whole slide. Depending on the scanner, it is possible to digitize bright‐field specimens as well as immunohistochemical stains and special stains.^1^ Scan duration varies dependent on specimen size, resolution, and device model. Usually, it ranges from several minutes to more than ten minutes per slide. In addition, modern scanners allow for automatic sequential scanning of larger sample series. Accordingly, dozens to hundreds of slides can be processed without manual intervention, for example, in continuous mode or overnight.

Because of the high resolution, the resulting image files have a large data volume. Given that uncompressed WSI files may be several gigabytes in size, low‐loss or lossy compression methods are usually used in practice. Various proprietary and open file formats are still in use, complicating standardized archiving and utilization across systems.^2^

### Viewer systems and digital diagnosis

Special viewer software solutions are used for viewing digitized slides. These allow for continuous zooming, moving, and marking of areas of interest as well as comparison of several sections or stains side by side on the screen. Depending on the system, viewers can be installed locally or operated in a web‐based manner. Given that presentation in true colors is essential for diagnostic use, calibration of monitors and sufficient screen resolution are of crucial importance.^6^ Moreover, digital diagnosis requires adjustment of ergonomic aspects. Studies have shown that screen size, resolution, and workplace design affect the duration of diagnosis and fatigue, especially in the case of complex dermatopathological questions with a high level of morphological details.^4^

### IT infrastructure and data storage

A high‐performance IT infrastructure is essential for the routine use of digital pathology systems. Apart from sufficient computing power, storage solutions with high capacity and data integrity are required, in particular. Depending on the size of the laboratory, several terabytes of image data are generated per year. The data can be stored locally, on a server, or in hybrid systems. While cloud‐based solutions are increasingly discussed, they require special data protection and organizational measures.^7^

The digital slides are accessed via secure networks and user management systems. Temporary access rights may be granted for consultations and second opinions, facilitating interdisciplinary exchange and resulting in many cases in elimination of physical transport routes that are replaced by digital data transmission.^5^

### Integration of image analysis and AI

Digital slides form the basis of computer‐aided image analysis and AI‐based assistance systems. These applications directly access WSI data and analyze defined image parameters, such as cell number, staining intensity, or morphological patterns. Technically, such systems require standardized image quality and consistent staining protocols, given that deviations may affect the analytical results.^3^, ^9^

Currently, these techniques are used mainly complementary to medical diagnosis. There is no intention for full automation of diagnostic decisions. Rather, the focus is on support, quantification, and reproducibility.^8^

While AI‐based assistance systems can support the dermatopathological diagnosis, they do not replace the medical diagnosis.

### Summary of technical prerequisites

Prerequisite for the successful implementation of digital pathology systems is the interplay of scanner technology, viewer software, IT infrastructure, and standardized workflows. Especially in dermatopathology, high image quality is essential for the reliable assessment of the fine morphological details. Accordingly, careful technical planning forms the basis for a reliable and efficient digital diagnostic workup.

Whole‐slide imaging enables high‐resolution acquisition of complete histological sections and forms the technical basis of digital pathology.

Prerequisites for a reliable digital diagnostic workup are consistent image quality, suitable viewer systems, and high‐performance IT infrastructure.

## WORKFLOW AND IMPLEMENTATION

Implementation of digital pathology systems in diagnostic routine requires adjustment of existing workflows and close coordination between pathology, IT department, and clinical partners. The aim is a seamless integration of digital processes into the established histological workflow without compromising diagnostic quality or efficiency.^7^, ^8^

Generally, the digital workflow starts after conventional histological processing. Following sectioning, staining, and mounting of specimens, the glass slides are fed to the WSI scanner. The digitized sections are then available for diagnosis via the viewer system. Depending on the laboratory structure, digital diagnosis may be performed parallel to the diagnosis by examination of glass slides or could gradually replace the latter. In many institutions, a transfer phase with hybrid workflows has been established in order to ensure diagnostic reliability and promote acceptance.^1^

A key aspect of implementation is the selection of suitable scanner and software systems. Apart from image quality and scan performance, aspects like ease of use, interfaces to laboratory information systems (LIS), and long‐term maintenance play a crucial role. Integration of digital slides in existing LIS structures allows for direct linkage of image data, findings, and clinical information and supports efficient processing of the case.^2^

Diagnosis of digital images differs in some points from conventional microscopy. Studies have shown that duration of diagnosis and navigation strategies change initially, but stabilize with increasing experience. Training courses and standardized introductory programs are, therefore, essential to ensure safe and efficient use of digital systems.^4^ Moreover, digital pathology systems facilitate dermatopathological consultations and second opinions. Based on the secure access to digital slides, external experts can be involved in a timely manner without shipment of physical slides. This shortens processing times and reduces the risk of loss or damage of slides.^5^

Additional aspects of implementation are archiving and long‐term availability of digital slides. Digital archives enable fast access to previous findings and support follow‐up, especially in case of chronic inflammatory skin diseases or recurrent skin tumors. At the same time, they require clear regulations concerning storage strategy, data storage, and access rights.^7^

Another relevant aspect is the interoperability of digital pathology systems. Different vendors use, in part, proprietary file formats and software solutions, which may complicate the exchange between systems and a later change of vendors. This may result in a so‐called vendor lock‐in situation, creating dependence on one single vendor where a system change is only possible with substantial technical and organizational effort. Open standards and compatible interfaces are, therefore, crucial to ensure long‐term data availability and flexible system integration.

The gradual implementation of digital pathology has turned out to be a feasible approach. Pilot projects with selected case groups and subdisciplines allow for early identification of technical and organizational challenges. This forms the basis for adjustment and standardization of workflows before completing the transition.^6^

In summary, the implementation of digital pathology is a multidisciplinary process taking technical, organizational, and personnel aspects equally into account. Structured planning and continuous evaluation are crucial for a successful implementation in clinical routine.

The implementation of digital pathology requires structured adjustment of existing workflows and close coordination between pathology and IT.

Hybrid workflows with parallel diagnosis by examination of glass slides and digital diagnosis have been established as a feasible transition in the implementation phase.

## QUALITY ASSURANCE AND VALIDATION

The implementation of digital pathology systems into diagnostic routine requires structured quality assurance and formal validation to ensure diagnostic performance equivalent to conventional diagnosis by examination of glass slides. International recommendations emphasize that digital systems have to be verified under real work conditions prior to routine use.^1^, ^6^

Generally, validation involves comparison of digital slides with the corresponding glass slides across a representative spectrum of cases. For this purpose, different diagnostic categories, stains, and levels of difficulty should be considered. The aim is to capture the diagnostic concordance as well as potential systematic discrepancies. Studies have shown that a high concordance between digital and conventional diagnosis can be achieved in case of adequate image quality and structured implementation.^3^, ^5^

An essential component of quality assurance is the continuous monitoring of technical parameters including scanner performance, accuracy of focus, color rendering, and monitor quality. Regular maintenance of the equipment and calibration of monitors are required to ensure consistent image quality. Changes in staining protocols or quality of sections may directly affect digital presentation and should, therefore, be integrated into existing measures of quality assurance.^2^

Moreover, organizational aspects play a central role. Clear regulations concerning release of diagnostic report, documentation, and archiving are required to ensure transparency of diagnostic decisions. Digital access to specimens must be executed via secure user management systems, especially in case of consultations and external second opinions.^7^

Additional requirements apply to the use of AI‐based assistance systems. These concern, in particular, clinical validation, regular performance review, and transparent presentation of application limits. Such systems should always be used under medical supervision and should be clearly differentiated from the independent diagnostic decision.^3^, ^8^

Overall, structured quality assurance forms the basis for a safe and reliable use of digital pathology systems in clinical routine.

Prior to routine use, digital pathology systems must be validated under real working conditions to ensure diagnostic equivalence to diagnosis by examination of glass slides.

## ORDER ENTRY AND DIGITAL REQUEST

Digital pathology is increasingly being integrated as early as the stage of requesting histopathological examinations. Electronic order entry systems allow for the structured recording of clinical questions, collection sites, and relevant additional information already before histological processing. This information is immediately available for pathology in digital form and can be integrated directly into the further diagnostic workflow.

The standardized digital request reduces media disruptions and improves transparency of diagnostic processes. Given that multiple biopsies and serial collections are frequently required in dermatology in particular, a structured order entry solution may contribute to clarity and allocation of specimens. Linking order entry, laboratory information system, and digital slides will also support consistent documentation.

Implementation of digital order entry systems requires close coordination between sending institutions and pathology. Uniform file formats, clear responsibilities, and adjustment of existing workflows are prerequisites for a seamless use. The diagnostic benefit is predominantly the result of the improved availability of information, not of the automation of the diagnostic decision.

However, the implementation of digital order entry systems involves challenges, caused in particular by inconsistent interfaces, incomplete clinical data, and variable acceptance in daily practice.

Digital order entry systems may support the pathological workflow, but they depend, however, on the quality of clinical data, compatible interfaces, and acceptance in clinical routine.

## LEGAL, LIABILITY AND BILLING‐RELATED ASPECTS

The implementation of digital pathology systems into clinical routine is not only associated with technical and organizational requirements but poses also questions with respect to legal, liability, and billing‐related aspects. Currently, the respective framework conditions have not yet been finally clarified and are subject to continuous development.

From a legal point of view, the responsibility for the assessment of digital slides is of particular relevance. When using digital systems and AI‐based assistance tools, the diagnostic responsibility remains with the physician releasing the diagnostic report. The use of supporting software does nothing to alter the medical liability for diagnostic decisions. Accordingly, clear internal regulations concerning release of the diagnostic report and documentation are required.

Concerning liability aspects, there is currently no consistent jurisdiction on the utilization of digital pathology systems and AI‐based applications in routine diagnostics. This applies, in particular, to questions of co‐responsibility in software‐aided analyses and the handling of technical malfunctions. Therefore, careful validation, documentation, and quality assurance are of crucial importance for minimizing potential risks.

Similarly, billing‐related aspects have not yet been conclusively regulated. Currently, the use of digital pathology systems and AI‐based analyses has not yet been included nationwide in existing billing systems. This applies to both remuneration of digital diagnosis and additional services in the context of digital consultations or computer‐aided evaluations. Therefore, the specific billability may vary dependent on area of care and regional regulations.

In summary, legal, liability, and billing‐related questions constitute a crucial parameter for the introduction of digital pathology. These factors should be considered during implementation and adjusted continuously to current legal developments.

Currently, the legal, liability, and billing‐related framework conditions of digital pathology have not yet been fully clarified and must be considered during implementation.

## ARTIFICIAL INTELLIGENCE IN DIGITAL PATHOLOGY

AI is an important enhancement of digital pathology systems and is based on computer‐aided analysis of digitized histological slides. The basis is formed by whole‐slide images that are analyzed by means of machine‐learning techniques. The aim of these applications is to support diagnostic processes, facilitate quantitative analyses, and increase reproducibility of histopathological findings.^3^, ^8^

In dermatopathological diagnosis, AI‐based systems are currently predominantly used as assistance tools. Typical fields of application are detection of suspicious areas, classification of histological patterns, and quantification of immunohistochemical markers. Especially in time‐consuming evaluations this may support the medical diagnosis. A prerequisite is a standardized image quality, given that variations in staining, section thickness, or scanner parameters may affect the analytical results.^8^, ^9^

Several studies have demonstrated that AI‐based procedures may achieve a high diagnostic performance in selected applications. At the same time, limitations exist with respect to transferability to different lab conditions and patient collectives. Given that the performance of such systems is largely dependent on underlying training data, careful clinical validation is required.^8^ Regulatory and legal aspects are relevant for their use in clinical routine. AI‐based applications are subject to requirements of the medical device regulation and must be approved and monitored accordingly. Moreover, a clear differentiation between supportive analysis and medical decision‐making is required. Accordingly, AI is currently used complementary to medical diagnosis and does not replace it.^3^ Apart from the diagnostic performance, comprehensibility and transparent presentation of algorithmic results are essential for clinical acceptance of AI‐based procedures. Another aspect relates to the integration of AI systems into existing digital workflows. Technically, the seamless integration of AI applications into viewer and laboratory information systems is required for their efficient use. From an organizational view, this requires clear responsibilities, especially with respect to interpretation of results and release of diagnostic reports.^6^ In the future, AI‐based systems might contribute to further standardization of dermatopathological diagnoses and allow for quantitative evaluations on a larger scale. Currently however, a critical evaluation of potential applications and limitations remains essential to ensure their appropriate use in clinical practice.^2^

AI‐based applications in digital pathology support the analysis of digital slides and require careful clinical validation and medical supervision.

## APPLICATIONS OF DIGITAL PATHOLOGY IN DERMATOLOGY

Digital pathology is used in dermatology and dermatopathology in different diagnostic contexts. Complete digitization of histological slides allows for efficient handling of dermatological questions, comparable documentation of findings, and support of interdisciplinary processes. The applications range from inflammatory dermatoses via pigmented lesions to epithelial skin tumors and immunohistochemical examinations^2^, ^5^ (Table 1).

**TABLE 1:** Key applications of digital pathology in dermatology and their clinical relevance.

Application	Benefits of digital pathology
Inflammatory dermatoses	Comparison of digital archives, follow‐up, standardized diagnostic workup
Melanocytic lesions	Digital consultations, parallel examination of serial sections and IHC
Epithelial skin tumors	Assessment of tumor architecture, resection margins, and recurrences
Immunohistochemistry	Parallel view of markers, supportive quantification
Consultations and second opinions	Location‐independent access, prompt professional feedback
Education and further training	Digital case archives, structured knowledge transfer

### Inflammatory dermatoses

In inflammatory skin diseases, the evaluation of subtle histomorphological changes plays a crucial role. Digital slides allow for a structured diagnosis and comparison of current findings with previous slides of the same patient. Especially for chronic, recurrent dermatoses, access to digital archives may facilitate follow‐up. Moreover, digital systems support standardized documentation of typical histological patterns and promote consistent diagnosis within larger institutions.^4^

### Melanocytic lesions

The diagnostic workup of melanocytic lesions places high demands on recognition of morphological details and assessment of architecture‐related criteria. Digital pathology allows for simultaneous observation of several section planes and additional immunohistochemical staining on one screen. Studies have shown that comparable diagnostic reliability between digital and conventional diagnosis can be achieved provided the image quality is adequate.^1^ Digital consultations are particularly important in case of diagnostically challenging lesions, and support professional exchange between specialized centers.

### Epithelial skin tumors

In epithelial skin tumors, such as basal cell carcinoma and squamous cell carcinoma, digital pathology enables efficient evaluation of tumor architecture, infiltration depth, and resection margins. Moreover, digital archiving facilitates comparison of primary tumors and recurrences. In surgical dermatology, prompt digital access to findings can contribute to therapy planning, especially in cases with complex or multiple lesions.^5^

### Immunohistochemistry and additional analyses

Digital systems offer advantages for evaluation of immunohistochemical staining. For fluorescence‐based staining there is the additional benefit that digital slides will not bleach contrary to physical material. Options for standardized rendering and the opportunity of viewing several markers side by side may facilitate interpretation. In addition, digital slides form the basis for computer‐aided quantifications, for example, in the case of proliferation markers or expression analyses. This requires consistent staining quality and a validated technical environment.^3^, ^9^

### Consultations, second opinions, and networking

An important application of digital pathology is the conduction of dermatopathological consultations. Given that digital slides can be made available promptly and independently of location, physical transport routes are eliminated and replaced by digital data transmission, usually resulting in significantly shorter transmission times. This promotes networking between dermatology offices, hospitals, and specialized centers and contributes to quality assurance, especially in rare or complex diseases.^7^

### Education and further training

Apart from routine diagnostics, digital pathology plays an increasing role in education and further training. Digital case archives enable structured access to typical and rare diagnoses and support standardized teaching of dermatopathological content. Especially against the background of limited availability of glass slides, this aspect is gaining in importance.^6^

In summary, digital pathology offers a wide range of potential applications in dermatology supporting diagnostic processes and promoting interdisciplinary exchange.

In dermatopathology, digital pathology supports consultations, follow‐up, and standardized documentation of findings, in particular.

Given the fine morphological details, digital evaluation of melanocytic lesions requires high image quality and careful quality assurance.

## ADVANTAGES AND LIMITATIONS OF DIGITAL PATHOLOGY

While digital pathology offers numerous advantages for dermatological diagnostics, it is also associated with technical, organizational, and structural limitations. A realistic assessment of both aspects is essential for appropriate use in clinical routine.^1^, ^2^

### Advantages

A major advantage of digital pathology is the location‐independent availability of histological slides. Digital slides can be analyzed in a time‐ and location‐independent manner, which is particularly important for dermatopathological consultations and second opinions. As a result, transport times of physical slides are avoided and processing times are shortened.^5^, ^7^

Digital archiving of histological slides enables rapid access to previous findings and facilitates follow‐up. This is particularly relevant for chronic inflammatory dermatoses, melanocytic lesions, and recurrent skin tumors. Moreover, digital documentation supports a standardized diagnostic workup and contributes to comparability of diagnostic decisions.^4^

In addition, digital slides form the basis of computer‐aided image analysis and AI‐based assistance systems. These may support quantitative evaluations and contribute to reproducibility of diagnostic parameters. A high diagnostic performance of such systems has been demonstrated in selected applications provided there is adequate technical and clinical validation.^3^, ^8^

Another advantage is the use of digital pathology for education and further training. Digital case archives enable a structured access to typical and rare dermatopathological findings and support standardized knowledge transfer irrespective of the availability of physical slides.^6^

### Limitations

Major limitations include the high demands on technical infrastructure and data storage. Digitization of high‐resolution slides results in considerable volumes of data requiring high‐performance storage solutions and stable network infrastructure. Especially for smaller institutions, this may present a challenge.^2^

Technical factors, such as scanning artifacts, focus problems, or insufficient color rendering may compromise diagnostic interpretation.

Another limiting aspect is that polarized light microscopy cannot be used for digital slides. This may impede analysis of certain diagnostic questions, such as identification of amyloid (Congo red) or assessment of foreign material. While experimental approaches for polarization‐sensitive digital imaging exist, a respective function has not yet been established in common routine scanners. Accordingly, complementary analysis by light microscopy may be required in these cases.

Regular quality assurance and calibration of scanners and monitors is, therefore, imperative. Moreover, digital diagnosis may initially be accompanied by a changed duration of the diagnosis until sufficient routine in handling of the systems has been achieved.^4^, ^6^ Similarly, limitations exist for the use of AI‐based systems. These involve, in particular, reliance on training data, limited generalizability, and regulatory requirements. Currently, AI applications are designed as supportive tools and do not replace medical diagnosis.^6^, ^9^ Overall, use of digital pathology requires careful consideration of benefits and limitations as well as a structured implementation that takes the technical and organizational framework into account.

While digital pathology offers opportunities for networking, standardization, and education, it requires careful technical and organizational implementation.

### Differentiation: Digital pathology and telepathology

Although the terms digital pathology and telepathology are sometimes used synonymously in clinical routine, they describe different concepts. Digital pathology refers to the complete digitization of histological slides by whole‐slide imaging allowing for a primarily digital diagnosis on the computer monitor. In contrast, telepathology refers to the transmission of pathological image information across locations with the aim of remote diagnosis or consultation.^1^ Applications of telepathology may be based on both static single images and live microscopy or digital whole slides. In this context, digital pathology constitutes the technical basis of modern telepathological concepts, given that it enables standardized, high‐resolution, and reproducible image transmission. In dermatopathological practice both approaches are particularly relevant for consultations and second opinions. However, they differ with respect to technical requirements, image quality, and diagnostic significance.^6^, ^7^

### Special dermatopathological aspects of digital diagnosis

The use of digital pathology in dermatopathology is associated with specific requirements. Evaluation of melanocytic lesions requires exact presentation of architectural and cytological details across several section planes. Digital systems allow for parallel viewing of serial sections and immunohistochemical stains, but require high image quality and consistent staining protocols.^4^ Pigment, cutting artifacts, or focusing issues may impair digital assessment of individual areas and require critical evaluation of the scanning quality. Especially in the case of highly pigmented lesions or very subtle epidermal changes, careful technical quality assurance is essential.^1^, ^5^ Furthermore, digital archiving of serial sections becomes increasingly important in dermatopathology, given that it facilitates follow‐up and comparative diagnoses. These aspects underscore that digital pathology must meet special morphological and technical requirements in dermatopathology in order to ensure its reliable diagnostic application.^2^

### Cognitive aspects of digital diagnosis

Digital diagnosis of histological slides is increasingly conducted in virtual work environments and is often associated with high case numbers and prolonged screen times at work. These conditions may increase cognitive load and affect the risk of fatigue effects. The concept of decision fatigue, in particular, describes the deteriorating quality of decisions with prolonged duration of diagnosis and increasing case numbers which may enhance the diagnostic vulnerability in routine operations.^10^ Apart from pure decision fatigue, the concept of cognitive robustness becomes increasingly important in dermatopathology. It describes the ability to maintain a stable diagnostic quality even under conditions of high work density, repetition, and digital overload. Depending on workflow design, break management, and individual experience, digital work environments can both support and compromise this robustness.^11^ Against this background, digital pathology should not be understood exclusively as a technical transformation but also as an alteration of cognitive working conditions. Aspects like rhythm of diagnosis, ergonomic design of digital workplaces, and deliberate strategies for reducing cognitive fatigue play a relevant role for diagnostic quality, especially for diagnosis of high numbers of routine cases on the screen.^10^, ^11^ In this context, high case numbers, legal uncertainty, and the aim of maximum security may favor defensive decision strategies and further increase the cognitive load in diagnostic routine.^12^ Experience from digital dermatopathological education has shown that virtual formats have gained a high level of acceptance, although they entail new load factors that should be considered when designing digital environments for working and diagnostic workup.^13^ Studies on implementation of digital pathology systems have shown that the transition from microscopic diagnosis of glass slides to digital diagnosis is perceived as demanding by many pathologists and dermatopathologists. Apart from technical aspects, individual learning curves, changed methods of perception and working, and questions of acceptance are particularly important. Several studies describe that confidence in digital diagnosis develops only in the course of structured implementation and validation processes.^1^, ^4^

Digital routine diagnostics on the screen may be accompanied with increased cognitive load; decision fatigue and individual cognitive robustness have a major effect on diagnostic quality.

## FUTURE PERSPECTIVES AND OUTLOOK

Digital pathology is in a dynamic process of development that will probably result in further integration into dermatological routine diagnostics in the coming years. Technical progress in scanner technology, IT infrastructure, and image processing will contribute to higher performance and broader application of digital systems.

A central area of development is the increasing establishment of AI‐based assistance systems. In the future, it is expected that such applications find increasing use in standardized quantification of histological parameters, for example, evaluation of proliferation markers or objective assessment of morphological patterns. Careful clinical validation and transparent presentation of performance limits and areas of applications will remain prerequisites. These systems will still be used complementary to medical diagnosis. Systematic reviews have shown that AI‐based classification models in skin cancer diagnosis can sometimes achieve equivalent or superior performance compared to human experts in experimental settings. At the same time, these reviews indicate that many studies were conducted under greatly simplified conditions and that transferability to clinical routine remains limited, especially for histopathological whole‐slide specimen.^14^

Another focus is the improved networking between dermatology offices, hospitals, and pathology institutes. Digital platforms enable a structured exchange of histological findings and promote interdisciplinary consultations. Especially in regions with limited availability of specialized dermatopathological expertise, this may contribute to quality assurance.^5^, ^7^

Moreover, digital pathology opens up perspectives for the combination of histological image data with clinical, molecular, and genetic information. In the future, such multimodal approaches may contribute to a more precise classification of dermatological diseases. Currently, corresponding concepts are mainly in the research stage and require standardized data formats and interoperable systems. Further relevance of digital pathology systems is anticipated for education and further training. Digital teaching archives and virtual case conferences enable location‐independent access to dermatopathological content and support standardized knowledge transfer.^6^, ^13^

In conclusion, digital pathology has the potential to support and structure diagnostic processes in dermatology in the long term. Continued successful development will, however, require that technical innovations go hand in hand with clear quality standards, validated applications, and defined responsibilities.

## CONFLICT OF INTEREST STATEMENT

None.

## [CME Questions – Lernerfolgskontrolle]

1. Was bezeichnet der Begriff *digitale Pathologie* korrekt?
  - Die Speicherung ausgewählter histologischer Einzelbilder.
  - Die Fernbefundung histologischer Präparate per Videomikroskopie.
  - Die vollständige Digitalisierung histologischer Präparate zur primär digitalen Befundung.
  - Die digitale Archivierung histologischer Befunde ohne Nutzung digitaler Präparate.
  - Die automatisierte histologische Diagnose ohne ärztliche Beteiligung.
2. Welche Technologie bildet die technische Grundlage der digitalen Pathologie?
  - Fluoreszenzmikroskopie.
  - Telekonferenzsysteme.
  - *Whole‐Slide‐Imaging*.
  - Molekulargenetische Analyse.
  - Virtuelle Realität.
3. Welche Voraussetzung ist für eine diagnostisch sichere digitale Befundung besonders wichtig?
  - Verwendung ausschließlich verlustfreier Bildformate.
  - Konstante Bildqualität und kalibrierte Monitore.
  - Hohe Internetgeschwindigkeit allein.
  - Vollständige Automatisierung der Befundung.
  - Verzicht auf Glasobjektträger.
4. Welcher Ansatz hat sich bei der Einführung digitaler Pathologie in der Routine bewährt?
  - Sofortige vollständige Umstellung ohne Übergangsphase.
  - Hybrider Workflow mit paralleler Glas‐ und Digitalbefundung.
  - Ausschließliche Nutzung digitaler Präparate für Konsile.
  - Einführung nur für immunhistochemische Färbungen.
  - Nutzung ausschließlich zu Lehrzwecken.
5. Was ist ein zentrales Ziel der Validierung digitaler Pathologiesysteme?
  - Verkürzung der Befundzeit.
  - Sicherstellung der diagnostischen Gleichwertigkeit zur Glasbefundung.
  - Reduktion der Speicheranforderungen.
  - Erhöhung der Automatisierungsrate.
  - Vereinfachung der Abrechnung.
6. In welchem Bereich bietet die digitale Pathologie in der Dermatologie einen besonderen Nutzen?
  - Ausschließlich bei infektiösen Dermatosen.
  - Bei Konsilen, Verlaufskontrollen und standardisierter Dokumentation.
  - Nur bei seltenen genetischen Erkrankungen.
  - Nur in der Forschung.
  - Ausschließlich bei intraoperativen Schnellschnitten.
7. Welche besondere Anforderung besteht bei der digitalen Beurteilung melanozytärer Läsionen?
  - Verzicht auf serielle Schnitte.
  - Verwendung geringerer Auflösung.
  - Hohe Bildqualität und sorgfältige Qualitätssicherung.
  - Ausschluss immunhistochemischer Färbungen.
  - Einsatz ausschließlich automatischer KI‐Systeme.
8. Welche Aussage zum Einsatz künstlicher Intelligenz in der digitalen Pathologie trifft zu?
  - KI ersetzt die ärztliche Befundung.
  - KI darf ohne klinische Validierung eingesetzt werden.
  - KI dient der unterstützenden Analyse unter ärztlicher Kontrolle.
  - KI ist nur für Forschungszwecke zulässig.
  - KI liefert auch bei niedriger Bildqualität zuverlässige Diagnosen.
9. Welche Limitation ist mit der digitalen Pathologie verbunden?
  - Fehlende Einsatzmöglichkeiten in der Dermatologie.
  - Hohe Anforderungen an IT‐Infrastruktur und Datenspeicherung.
  - Grundsätzlich unzureichende diagnostische Aussagekraft.
  - Ausschluss der Nutzung für Lehre.
  - Unmöglichkeit der Archivierung.
10. Welche Aussage zu rechtlichen und abrechnungstechnischen Aspekten ist korrekt?
  - Diese sind vollständig geklärt und einheitlich geregelt.
  - Die Haftung geht bei digitaler Befundung auf den Softwarehersteller über.
  - Digitale Pathologie ist in allen Bereichen gesondert abrechenbar.
  - Rechtliche, haftungsrechtliche und abrechnungstechnische Rahmenbedingungen sind derzeit nicht vollständig geklärt.
  - Digitale Pathologie ist unabhängig von wirtschaftlichen und abrechnungstechnischen Aspekten implementierbar.

Liebe Leserinnen und Leser, der Einsendeschluss an die DDA für diese Ausgabe ist der 30. Oktober 2026.

Die richtige Lösung zum Thema Komplikationen von Laser und Energie‐basierten Systemen in der Dermatologie: Klassifikation, Management und Prävention in Heft 3/2026 ist: 1b, 2c, 3c, 4b, 5b, 6b, 7a, 8d, 9d, 10a

Bitte verwenden Sie für Ihre Einsendung das aktuelle Formblatt auf der folgenden Seite oder aber geben Sie Ihre Lösung online unter http://jddg.akademie-dda.de ein.
